# Low-Dose Glyphosate Exposure Alters the Enteric Tachykinin System in the Porcine Small Intestine

**DOI:** 10.3390/ijms27198598

**Published:** 2026-09-25

**Authors:** Zofia Walczak-Mośny, Małgorzata Chmielewska-Krzesińska, Barbara Jana, Katarzyna Palus

**Affiliations:** 1 Department of Clinical Physiology, Faculty of Veterinary Medicine, University of Warmia and Mazury in Olsztyn, Oczapowskiego St. 13, 10-718 Olsztyn, Poland; zofia.walczak@doktorant.uwm.edu.pl; 2 Department of Pathophysiology, Forensic Veterinary and Administration, Faculty of Veterinary Medicine, University of Warmia and Mazury in Olsztyn, Oczapowskiego St. 13, 10-718 Olsztyn, Poland; malgorzata.chmielewska@uwm.edu.pl; 3 InLife Institute of Animal Reproduction and Food Research, Polish Academy of Sciences, 18 Trylińskiego St., 10-683 Olsztyn, Poland; b.jana@pan.olsztyn.pl

**Keywords:** glyphosate, enteric nervous system, substance P, neurokinin receptors, neuroplasticity, porcine model, small intestine

## Abstract

Glyphosate is a widely used herbicide, yet its effects on the enteric nervous system (ENS) remain poorly understood. This study investigated the effects of low-dose glyphosate exposure on substance P (SP)-immunoreactive neurons and tachykinin receptor gene expression in the porcine small intestine. Fifteen juvenile pigs were assigned to three groups (*n* = 5 per group): a control group and two groups receiving oral glyphosate for 28 days at doses corresponding to the estimated maximum daily intake in Europe (0.05 mg/kg BW/day) and the acceptable daily intake (0.5 mg/kg BW/day). Double immunofluorescence was used to determine the percentage of SP-immunoreactive neurons in the myenteric (MP), outer submucosal (OSP), and inner submucosal (ISP) plexuses of the duodenum, jejunum, and ileum. The expression of *TACR1*, *TACR2*, and *TACR3* was analysed by RT-qPCR. Glyphosate exposure increased the percentage of SP-immunoreactive neurons in all analysed intestinal segments and plexuses, with a clear dose-related pattern. *TACR2* mRNA expression was significantly increased in the duodenum in both glyphosate-exposed groups but decreased in the ileum at the higher glyphosate dose. *TACR3* mRNA expression was significantly decreased in the duodenum in both exposed groups and in the jejunum at the higher dose, whereas *TACR1* expression remained unchanged. SOD activity was significantly increased in the duodenum and ileum of pigs exposed to the higher glyphosate dose, whereas no significant changes were observed in the jejunum. These findings demonstrate that low-dose glyphosate exposure is associated with region-specific alterations in selected components of the enteric tachykinin system, indicating a complex neurochemical response of the ENS to glyphosate.

## 1. Introduction

Glyphosate is a synthetic, non-selective herbicide that serves as the active ingredient in glyphosate-based herbicides (GBH), widely used in agriculture for weed control [1]. This compound belongs to the phosphonate group and is highly soluble in water, which facilitates its application as a spray, but also promotes its spread within the environment. Consequently, glyphosate is detected in the soil, surface water and groundwater, atmospheric precipitation, as well as certain food products [2].

The mechanism of glyphosate action involves inhibition of 5-enolpyruvylshikimate-3-phosphate synthase (EPSPS), a key enzyme in the shikimate pathway that is responsible for the biosynthesis of aromatic amino acids in plants and certain microorganisms [3]. Initially, glyphosate was considered relatively safe for vertebrates, as this metabolic pathway is absent in animal cells. Currently, however, there is growing attention on the presence of the shikimate pathway in many bacteria and fungi that colonise animal bodies, including those found in the intestinal microbiota. This may represent an indirect mechanism by which glyphosate affects the host [4]. Furthermore, the literature has described a number of changes at the cellular and tissue levels, observed after exposure to glyphosate or GBH, including, *inter alia*, disturbances in liver and kidney function, increased oxidative stress, and modifications in the inflammatory response [5,6]. Reports on carcinogenic and neurotoxic effects remain partially ambiguous and are largely determined by the research model, the dose, and the formulation composition. At the same time, experimental studies have documented the ability of glyphosate and certain GBH formulations to induce neurotoxic changes, including disturbances in neuronal transmission, neuroinflammation, and oxidative stress [7,8]. Furthermore, some long-term studies on animal models have demonstrated an increased incidence of certain neoplasms, which suggests a potential carcinogenic effect under specific exposure conditions [9].

An increasing number of studies are focusing on the effects of glyphosate on the gastrointestinal tract. Animal models have demonstrated that exposure to this compound can alter the composition of the intestinal microbiota, including a decrease in potentially beneficial bacterial populations and a relative increase in microorganisms with pro-inflammatory potential [10]. These changes can be accompanied by a weakening of the intestinal barrier integrity, manifested by reduced expression of tight junction proteins and an intensified local inflammatory response in the intestinal mucosa [11]. In addition to the epithelium and the microbiota, the enteric nervous system (ENS) is a significant component that responds to disturbances in gastrointestinal tract homeostasis. The ENS is an extensive network of neurons and glial cells capable of autonomously controlling motility, secretion, blood flow, and intestinal barrier function. At the same time, it remains in close interaction with the immune system and epithelial cells, integrating mechanical, chemical and inflammatory signals [12]. In response to injury, inflammation, or exposure to toxic agents, neurochemical remodelling may occur in the ENS, involving changes in the expression profile of neurotransmitters and neuropeptides without significant neuronal loss. This phenomenon is regarded as a manifestation of ENS plasticity, reflecting both adaptive mechanisms and the early stages of neuronal dysfunction [13].

Among the ENS neuropeptides, tachykinins are considered particularly significant, as they link the regulation of intestinal motility with neuro-immune mechanisms. Substance P (SP) is one of the main mediators of sensory signalling and neurogenic inflammation within the intestinal wall. This peptide promotes the activation of immunocompetent cells, modulates microvascular permeability, and may enhance local inflammatory responses in the intestinal mucosa [14,15]. Previous studies have shown that the neurochemical phenotype of enteric neurons may change in response to functional and inflammatory gastrointestinal disorders, exposure to toxic agents such as acrylamide or bisphenol A, and metabolic disturbances including streptozotocin-induced diabetes [16,17,18]. Substance P exerts its biological effects primarily through the neurokinin 1 (NK1) receptor, a G protein-coupled receptor. This receptor plays a significant role in regulating sensory transmission and inflammatory mechanisms within the wall of the gastrointestinal tract. To a lesser extent, substance P may also interact with NK2 and NK3 receptors, which modulate smooth muscle layer activity and signal processing in the local neural networks of the enteric nervous system [19,20].

The porcine gastrointestinal tract exhibits significant anatomical and functional similarities to that of humans, including a comparable structure of the intestinal wall layers and a well-developed ENS. The neurochemical organisation of the ENS in this species has been characterised in detail, making it suitable for assessing changes in the neurochemical phenotype of enteric neurons in response to toxic and inflammatory stimuli. For this reason, the pig is regarded as a valuable model for translational research into gastrointestinal pathophysiology [21].

Despite the growing number of studies on the effects of glyphosate and glyphosate-based herbicides (GBH) on the intestinal microbiota and the integrity of the intestinal barrier, as well as reports suggesting that exposure to glyphosate may modify the neurochemical phenotype of neurons in the enteric nervous system (ENS) in the pig [22,23,24], there is still a lack of data regarding its effects on the tachykininergic system. In particular, to date, it has not been determined whether low-dose exposure to glyphosate affects the population of substance P-immunoreactive neurons or the expression of neurokinin receptors in the small intestine. The present study aimed to assess the effects of oral exposure to low doses of glyphosate on the population of ENS neurons immunoreactive to substance P and the expression of NK1, NK2, and NK3 receptors in the porcine small intestine.

## 2. Results

### 2.1. Double Immunofluorescence Analysis of SP-Immunoreactive Neurons

Glyphosate exposure was associated with an increase in the percentage of SP-immunoreactive neurons in all analysed intestinal segments and enteric plexuses, although the magnitude and statistical significance of these changes varied depending on the dose and intestinal region (Table 1).

In the duodenum, the percentage of SP-immunoreactive (SP-IR) neurons increased with increasing glyphosate dose. In the MP, the highest value was observed in group G2 (5.55 ± 0.47%), which was significantly higher than in both the control group (1.22 ± 0.15%; *p* < 0.001) and group G1 (1.55 ± 0.24%; *p* < 0.001). Similar increases were observed in the OSP and ISP, with significantly higher values in group G2 than in both the control and G1 groups (Table 1, Figure 1).

In the jejunum, glyphosate exposure increased the percentage of SP-IR neurons in all three enteric plexuses. The increase was greater in group G2 than in group G1, with significant differences between the two exposed groups in the MP, OSP, and ISP (Table 1, Figure 2). The largest relative increase was observed in the ISP, where the percentage of SP-IR neurons reached 43.06 ± 1.21% in group G2 compared with 12.35 ± 0.70% in the control group (*p* < 0.001) (Table 1, Figure 2).

In the ileum, the percentage of SP-IR neurons was also increased following glyphosate exposure. In the MP, group G2 showed a significantly higher percentage of SP-IR neurons than the control group (6.11 ± 0.53% vs. 1.01 ± 0.19%; *p* < 0.001). Significant increases were also observed in the OSP and ISP, particularly in group G2 (Table 1, Figure 3).

Overall, a dose-related effect was observed across all analysed intestinal segments and enteric plexuses, with significantly higher percentages of SP-IR neurons in group G2 than in group G1.

In contrast, glyphosate exposure did not significantly affect the number of HuC/D-positive neurons per ganglion in any of the analysed intestinal segments or enteric plexuses (Table 2). Although minor numerical differences were observed between the control and glyphosate-treated groups in some plexuses, none of these differences reached statistical significance, and no consistent dose-dependent pattern was evident. The mean numbers of HuC/D-positive neurons per ganglion remained comparable among the C, G1, and G2 groups across the duodenum, jejunum, and ileum.

### 2.2. RT-qPCR Analysis of NK Receptor Expression

Glyphosate exposure resulted in receptor- and segment-specific changes in tachykinin receptor gene expression. Significant differences from the control group were observed for *TACR2* and *TACR3*, whereas *TACR1* expression remained unchanged (Figure 4).

*TACR1* (NK1) mRNA expression did not differ significantly between groups in any of the analysed intestinal segments. Transcript levels in both glyphosate-exposed groups were comparable to control values, with no apparent dose-related pattern (Figure 4).

*TACR2* (NK2) mRNA expression was significantly increased in the duodenum in both glyphosate-exposed groups compared with the control group (*p* < 0.001). In contrast, in the ileum, *TACR2* mRNA expression was significantly decreased in group G2 compared with the control group (*p* < 0.05), whereas no significant changes were observed in the jejunum. No significant differences were found between groups G1 and G2 in any of the analysed intestinal segments (Figure 4).

*TACR3* (NK3) mRNA expression was significantly decreased in the duodenum in both glyphosate-exposed groups compared with the control group (*p* < 0.001). In the jejunum, a significant decrease was observed in group G2 (*p* < 0.05), whereas the change in group G1 did not reach statistical significance. In the ileum, *TACR3* mRNA expression showed a downward tendency, but the differences did not reach statistical significance. No significant differences were observed between groups G1 and G2 (Figure 4).

### 2.3. Superoxide Dismutase (SOD) Activity

In the duodenum, SOD activity was 198.25 ± 2.15 U/mg protein in the C group, 201.65 ± 1.74 U/mg protein in the G1 group, and 221.65 ± 0.97 U/mg protein in the G2 group. One-way ANOVA revealed a significant overall effect of treatment (F(2,12) = 55.77, *p* < 0.001). Tukey’s post hoc test showed that SOD activity was significantly higher in the G2 group than in both the C and G1 groups (*p* < 0.001), whereas no significant difference was observed between the C and G1 groups (Figure 5).

In the jejunum, SOD activity was 204.54 ± 1.05 U/mg protein in the C group, 207.12 ± 1.56 U/mg protein in the G1 group, and 206.87 ± 1.97 U/mg protein in the G2 group. No statistically significant differences were observed between the groups (one-way ANOVA, F(2,12) = 0.82, *p* = 0.464) (Figure 5).

In the ileum, SOD activity was 176.76 ± 1.53 U/mg protein in the C group, 180.23 ± 0.89 U/mg protein in the G1 group, and 195.32 ± 1.82 U/mg protein in the G2 group. One-way ANOVA revealed a significant overall effect of treatment (F(2,12) = 45.36, *p* < 0.001). Tukey’s post hoc test showed that SOD activity was significantly higher in the G2 group than in both the C and G1 groups (*p* < 0.001), while the difference between the C and G1 groups was not significant (Figure 5).

## 3. Discussion

The results of the present study demonstrate that oral exposure of pigs to low doses of glyphosate induces marked neurochemical changes in the enteric nervous system of the small intestine, manifested by an increase in the percentage of substance P-immunoreactive neurons and is additionally associated with alterations in tachykinin receptor gene expression in the intestinal wall. In all analysed intestinal segments (duodenum, jejunum, and ileum), an increase in the percentage of SP-immunoreactive neurons was observed, with a clear dose-related pattern in the majority of cases. At the same time, receptor- and region-specific regulation of tachykinin receptor gene expression was observed. *TACR2* (NK2) mRNA expression was increased in the duodenum in both glyphosate-exposed groups but decreased in the ileum at the higher glyphosate dose. *TACR3* (NK3) mRNA expression was reduced in the duodenum in both exposed groups and in the jejunum at the higher dose. In contrast, *TACR1* (NK1) mRNA expression remained unchanged. The obtained results are consistent with previous reports indicating that the percentage of substance P-immunoreactive enteric neurons may increase in response to damaging, inflammatory, or toxic factors, which is interpreted as part of the adaptive neurochemical remodelling of the ENS [25,26,27,28].

To better understand the biological significance of the observed changes, they should be considered in the context of the established functions of substance P within the enteric nervous system. Substance P plays an important role in the transmission of visceral pain, the regulation of gastrointestinal motility, and the modulation of the inflammatory response [14,15]. From a neurogastroenterological perspective, it is also important to note that the tachykinin system is involved not only in the control of motility but also in the modulation of visceral sensation, which may be relevant to the development of visceral hypersensitivity following chronic exposure to environmental factors [15,29]. In this context, the increase in the proportion of SP-immunoreactive neurons may reflect enhanced SP expression and changes in the neurochemical phenotype of enteric neurons, indicating a plastic response of the ENS to glyphosate exposure. Such neurochemical remodelling may potentially contribute to the modulation of both motor and sensory functions of the gastrointestinal tract. Importantly, the increase in the proportion of SP-immunoreactive neurons was not accompanied by significant changes in the total number of HuC/D-positive neurons per ganglion in any of the analysed intestinal segments or enteric plexuses. This finding supports the interpretation that the observed increase in SP immunoreactivity is more likely related to changes in the neurochemical phenotype of enteric neurons rather than to a reduction in the overall neuronal population.

Analysis of tachykinin receptor gene expression in the intestinal wall provides additional information on the nature of the observed changes. Importantly, no parallel relationship was observed between the increase in the percentage of SP-positive neurons and changes in tachykinin receptor mRNA expression at the tissue level. Despite the marked increase in the percentage of SP-positive neurons, *TACR1* expression remained stable, whereas *TACR2* and *TACR3* showed receptor- and region-specific patterns of regulation. *TACR2* mRNA expression increased in the duodenum in both glyphosate-exposed groups but decreased in the ileum at the higher glyphosate dose, while *TACR3* expression decreased in the duodenum in both exposed groups and in the jejunum at the higher dose. No parallel pattern was observed between changes in neuronal SP immunoreactivity and tissue-level tachykinin receptor gene expression. However, this apparent dissociation should be interpreted cautiously because the two analyses were performed at different levels of biological organization: SP immunoreactivity was quantified specifically in enteric neurons, whereas *TACR* mRNA expression was assessed in full-thickness intestinal tissue containing multiple neuronal and non-neuronal cell populations. Therefore, the lack of a parallel relationship may, at least in part, reflect this difference in cellular and anatomical resolution rather than independent regulation of individual components of the tachykinin system. Of particular interest is the region-dependent regulation of *TACR2*, as NK2 receptors play an important role in the regulation of gastrointestinal motility, including intestinal smooth muscle contractions. It has been demonstrated that modulation of NK2 signalling affects intestinal motor responses, and disturbances in this signalling may alter the contractile function of the gastrointestinal tract [15,30]. The opposite direction of *TACR2* regulation in the duodenum and ileum further supports the concept of region-specific modulation of tachykinin signalling following glyphosate exposure [27,31]. The most consistent changes in tachykinin receptor expression were observed in the duodenum. One possible explanation is that the proximal small intestine may experience relatively high local exposure following oral administration of glyphosate, although this hypothesis was not directly tested in the present study [32].

Similar patterns of neurochemical changes have previously been described in various gastrointestinal pathologies, allowing the present findings to be considered within a broader biological context. In gastrointestinal diseases such as inflammatory bowel disease (IBD), DSS-induced models of intestinal inflammation, and inflammatory processes of infectious aetiology, increased expression of substance P and neurokinin receptors associated with activation of the neuroimmune axis has been demonstrated [28,33,34,35]. Similar changes have also been observed following exposure to environmental and dietary xenobiotics, including acrylamide and bisphenol A [17,36]. In both models, an increase in the proportion of SP-positive neurons and alterations in the neurochemical profile of the ENS were observed and interpreted as manifestations of adaptive plasticity of the enteric nervous system. In this context, the increase in SP-immunoreactive neurons observed in the present study may likewise reflect neuroplastic processes within the ENS, whereas the accompanying changes in tachykinin receptor expression should be interpreted at the level of the intestinal wall as a whole. An important novelty of the present study is, however, the demonstration that glyphosate exposure is associated not only with an increase in the SP-immunoreactive component of the ENS but also with selective, region-specific regulation of neurokinin receptor gene expression in the intestinal wall. In particular, the opposite trends in *TACR2* and *TACR3* expression suggest that the response to glyphosate exposure is complex and cannot be explained solely by an increase in substance P immunoreactivity.

Given the observed similarities to neurochemical changes reported under other pathological and toxic conditions, it is reasonable to consider the potential molecular mechanisms underlying the present findings. Although these mechanisms are not clearly defined, the available data point to several possible intermediate pathways. One of them is the induction of oxidative stress, which is well documented in studies of glyphosate exposure and involves increased production of reactive oxygen species and disturbances in antioxidant balance [6,37,38]. In the present study, SOD activity was significantly increased in the duodenum and ileum of pigs exposed to the higher glyphosate dose, whereas no significant changes were observed in the jejunum. As SOD represents a major component of the enzymatic antioxidant defense system, its increased activity may reflect a compensatory response to enhanced oxidative challenge rather than direct evidence of oxidative damage. The segment-specific pattern observed here further suggests that individual regions of the small intestine may differ in their susceptibility or adaptive response to glyphosate exposure. Oxidative stress may modulate the neurochemical phenotype of enteric neurons, including the expression of neuropeptides and may also influence tachykinin receptor expression in different cellular compartments of the intestinal wall [12]. Thus, alterations in antioxidant defense may constitute one of the mechanisms contributing to the changes observed in the enteric tachykinin system. However, this relationship cannot be established directly on the basis of SOD activity.

In addition to oxidative stress, modulation of the intestinal microbiota may also play an important role. Inhibition of the shikimate pathway in bacteria may disturb the composition of the intestinal microbiota, potentially promoting the development of a pro-inflammatory environment [10,39,40,41,42]. These changes may influence the enteric nervous system through the microbiota–gut–nervous system axis, including the actions of bacterial metabolites, immune signals, and alterations in intestinal barrier function [43,44,45].

Another potential mechanism contributing to the effects of glyphosate may involve activation of the immune response. Glyphosate exposure may therefore induce a multilevel response within the intestinal environment involving disturbances in redox homeostasis, changes in microbiota composition, and activation of immune mechanisms. These factors may jointly modulate neurotransmitter pathways involved in gastrointestinal motility, visceral sensation, and local inflammatory responses [42,46]. Glyphosate has been shown to influence the production of pro-inflammatory cytokines, including TNF-α, IL-1β, and IL-6 [10,47,48]. These mediators can affect enteric neurons and glial cells, leading to changes in neuropeptide expression, while changes in tachykinin receptor expression detected at the tissue level may additionally involve non-neuronal cell populations within the intestinal wall [31]. In this context, alterations in tachykinin signalling, particularly those involving the NK2 receptor, may represent a secondary response to local inflammatory processes.

The mechanisms described above are not mutually exclusive and may operate simultaneously, leading to a coordinated response within the intestinal environment, including the enteric nervous system, to glyphosate exposure. Oxidative stress, disturbances in the microbiota, and immune activation may therefore represent interconnected pathways capable of influencing enteric neurotransmitter systems as well as other cellular compartments of the intestinal wall. It cannot be excluded that the observed changes represent not only adaptive responses but also early manifestations of dysregulatory processes within the intestinal wall and the enteric nervous system.

Although several possible pathways underlying the effects of glyphosate on the enteric nervous system have been discussed, these mechanistic interpretations should be treated with caution. SOD activity was assessed as an indicator of antioxidant defense, however the present study did not include additional markers of oxidative damage or redox status. Therefore, the involvement of oxidative stress in the observed neurochemical changes cannot be conclusively established. The study also did not directly assess changes in intestinal microbiota composition, immune activation, or functional alterations in intestinal motility and sensation. This limits the possibility of establishing the mechanisms responsible for the observed neurochemical changes. A further limitation is that glyphosate and its major metabolite, AMPA, were not quantified in plasma or intestinal tissue. Therefore, the present study does not provide direct information on systemic exposure or tissue concentrations of glyphosate and AMPA at the administered doses. Another limitation concerns the cellular specificity of the RT-qPCR analysis. Because gene expression was assessed in homogenates of the intestinal wall containing multiple neuronal and non-neuronal cell populations, the observed alterations in *TACR2* and *TACR3* mRNA expression cannot be attributed specifically to enteric neurons. Tachykinin receptors may also be expressed by non-neuronal cell types within the intestine, including smooth muscle, epithelial, and immune cells. Therefore, the observed receptor expression patterns may reflect responses occurring in both neuronal and non-neuronal compartments. This methodological difference should also be considered when interpreting the apparent lack of parallel changes between neuronal SP immunoreactivity and tissue-level *TACR* expression. In addition, *TACR1*, *TACR2*, and *TACR3* expression was assessed only at the mRNA level and was not confirmed at the protein level. Therefore, it cannot be assumed that the observed transcriptional changes were accompanied by corresponding alterations in receptor protein abundance. It appears particularly important to determine whether alterations in neurokinin receptor gene expression are accompanied by changes in intestinal motor and sensory functions. Although the porcine gastrointestinal tract closely resembles that of humans, species-specific differences should be considered when extrapolating these findings. The relatively small number of animals per group may also limit the precision and generalisability of the observed effects. Furthermore, only female pigs were included in the present study. Given the reported sex-related differences in enteric nervous system neurochemistry and xenobiotic metabolism, the present findings cannot necessarily be extrapolated to males. Future studies including both sexes will be required to determine whether the observed responses to glyphosate are sex-dependent.

## 4. Materials and Methods

### 4.1. Approval of the Ethics Committee

All procedures were carried out in accordance with Directive 2010/63/EU of the European Parliament and of the Council on the protection of animals used for scientific purposes and the applicable Polish legislation on the protection of animals used for scientific or educational purposes. The experimental protocol was approved by the Local Ethics Committee for Animal Experimentation in Olsztyn (Approval No. 62/2020, approval date 21 October 2020).

### 4.2. The Animals and Experimental Design

The study was conducted on fifteen juvenile female Danish Landrace pigs (gilts) aged approximately eight weeks, with an average body weight of 20 kg. Females were selected to maintain a homogeneous experimental population and reduce biological variability related to sex. The use of sexually immature animals also minimized the potential influence of cyclic changes in ovarian hormone levels. Before the start of the experiment, the animals underwent a 7-day acclimatisation period under standard housing conditions. During the acclimatisation period and throughout the experiment, the animals were housed in groups under standard husbandry conditions, fed the same commercially available diet, and provided with water ad libitum. Samples of the feed were analyzed for glyphosate residues in an accredited analytical laboratory. Glyphosate concentrations were below the limit of quantification (LOQ; 0.01 mg/kg). Environmental enrichment was provided throughout the study. The animals were then randomly assigned to three equally sized experimental groups (*n* = 5 per group). The control group (C) received empty gelatine capsules once daily. Animals in the experimental groups received glyphosate (analytical standard, purity > 99.5%, Sigma-Aldrich, St. Louis, MO, USA, CAS No. 1071-83-6) orally in gelatine capsules once daily during the morning feeding for 28 consecutive days. The dose administered to group G1 corresponded to the estimated maximum daily intake in Europe (0.05 mg/kg BW/day), whereas the dose administered to group G2 corresponded to the acceptable daily intake (ADI; 0.5 mg/kg BW/day). The animals were weighed weekly, and the administered glyphosate doses were adjusted according to the current body weight. At the end of the 28-day exposure period, the animals were premedicated with azaperone (Stresnil, Janssen Pharmaceutica NV, Beerse, Belgium) at a dose of 4 mg/kg BW administered intramuscularly. After 15 min, euthanasia was performed by intravenous administration of sodium pentobarbital (Morbital, Biowet Puławy Sp. z o.o., Puławy, Poland; 0.6 mL/kg BW). Immediately after euthanasia, samples of the duodenum, jejunum, and ileum were collected. Tissue samples intended for immunofluorescence and molecular analyses were processed separately. Samples intended for immunofluorescence analysis were fixed in 4% paraformaldehyde in phosphate-buffered saline (PBS, pH 7.4) for 1 h at room temperature. The tissues were then rinsed in PBS for three days, cryoprotected in 30% sucrose solution, and stored at 4 °C until further processing. Samples intended for gene expression analysis were immersed in RNAlater^®^ solution (Ambion, Austin, TX, USA), maintained overnight at 4 °C, and subsequently stored at −80 °C until RNA isolation.

### 4.3. Double Immunofluorescence Staining

Cryostat sections 12 µm thick were prepared from each examined segment of the small intestine under investigation. To identify all neurons, an antibody against the HuC/D protein (mouse, Invitrogen, Waltham, MA, USA, Cat. No. A-21271, working dilution: 1:1000), a commonly used pan-neuronal marker, was used. To assess the presence of substance P, a primary antibody against SP (SP, rat, AbDSerotec, Raleigh, NC, USA, Cat. No. 8450-0505, working dilution: 1:150) was used in accordance with the established staining protocol [22]. The sections were incubated with the primary antibodies overnight.

After incubation with primary antibodies, species-specific secondary antibodies conjugated to Alexa Fluor 488 fluorochromes were used (donkey anti-mouse, ThermoFisher Scientific, Waltham, MA, USA; Cat. No. A21202; working dilution: 1:1000), and Alexa Fluor 546 (goat anti-rat, ThermoFisher Scientific, Waltham, MA, USA; Cat. No. A11081, working dilution: 1:1000) were applied. Incubation with the secondary antibodies was performed for 1 h. The specificity of the immunofluorescence reactions was verified by omitting the primary antibodies, performing substitution tests and conducting antigen pre-adsorption controls.

Substance P-immunoreactive neurons were identified within the myenteric plexus (MP) and the inner (ISP) and outer (OSP) submucosal plexuses. The number of SP-positive neurons was determined as a percentage of all HuC/D-positive neurons within each plexus. For each animal, at least 500 HuC/D-positive neurons were analysed in each plexus of each intestinal segment. The percentage of SP-immunoreactive neurons was calculated separately for each animal and used as a single biological replicate in the statistical analysis. Additionally, the number of neurons per ganglion was assessed in each plexus. In each experimental group, 50 ganglia of each plexus type (MP, OSP, and ISP) were analysed in each intestinal segment, corresponding to 10 ganglia per animal. The total number of HuC/D-positive neuronal cell bodies within each ganglion was counted. The investigators performing the immunofluorescence quantification were blinded to the experimental group allocation. To prevent the same cells from being counted multiple times, sections at least 100 µm apart were selected for analysis. The sections were analysed using an Olympus BX51 epifluorescence microscope (Olympus, Tokyo, Japan), and photographic documentation was obtained using a camera (Olympus XM 10) with fixed exposure settings and processed using Olympus Cell F image analysis software, version 1.16 (Olympus, Tokyo, Japan).

### 4.4. RT-qPCR Analysis of Tachykinin Receptor Gene Expression

Total RNA was isolated from approximately 50 mg of full-thickness intestinal tissue containing all layers of the intestinal wall, including the serosa, muscularis externa, submucosa, and mucosa. The isolation was performed using the GeneJET™ RNA Purification Kit (Thermo Fisher Scientific, Waltham, MA, USA) according to the manufacturer’s instructions. RNA purity and concentration were assessed spectrophotometrically using a NanoVue® spectrophotometer (GE Healthcare Bio-Sciences, Uppsala, Sweden). Reverse transcription was then performed to obtain complementary DNA (cDNA) using the Maxima First Strand cDNA Synthesis Kit for RT-qPCR (Thermo Fisher Scientific, Waltham, MA, USA). The cDNA synthesis reaction was carried out in a Biometra T Gradient Professional Basic thermal cycler (Biometra GmbH, Göttingen, Germany) according to the following programme: 10 min at 25 °C, 30 min at 65 °C, 5 min at 85 °C, followed by 10 min at 4 °C. The obtained cDNA samples were stored at −80 °C until further analysis.

The expression levels of *TACR1* (NK1), *TACR2* (NK2), and *TACR3* (NK3) were determined in each sample, with porcine *GAPDH* used as the reference gene. Primers were designed using the Primer-BLAST tool (National Center for Biotechnology Information, Bethesda, MD, USA; https://www.ncbi.nlm.nih.gov/tools/primer-blast/, accessed on 15 October 2020), and their sequences and GenBank accession numbers are listed in Table 3.

All RT-qPCR reactions were performed in technical triplicate on 96-well plates using the 7500 Fast Real-Time PCR System (Applied Biosystems, Waltham, MA, USA). The amplification programme comprised an initial denaturation step for 10 min at 95 °C, followed by 40 cycles of denaturation for 15 s at 95 °C and primer annealing and extension for 1 min at 60 °C. Relative gene expression was calculated using the 2^−ΔCt^ method, where ΔCt was calculated as the difference between the Ct value of the target gene and that of the reference gene (*GAPDH*). The results are presented as relative expression levels normalized to *GAPDH*.

### 4.5. Determination of Superoxide Dismutase (SOD) Activity

Samples of the duodenum, jejunum, and ileum were collected from the same animals as those used for the immunofluorescence analyses. For each intestinal segment, samples from five animals per experimental group were analyzed (*n* = 5/group). For preparation of intestinal homogenates, tissue fragments from each intestinal segment were collected from each animal in equal weight proportions and pooled to obtain one composite sample representative of the respective intestinal segment of that animal. Samples designated for biochemical analyses were stored at −80 °C until further processing.

Tissue samples designated for SOD determination were homogenized on ice in 20 mM HEPES buffer (pH 7.2) containing 1 mM EGTA, 210 mM mannitol, and 70 mM sucrose, using 10 mL of buffer per 1 g of tissue. The homogenates were centrifuged at 1500× *g* for 5 min at 4 °C, and the resulting supernatants were used for enzymatic analyses. SOD activity was determined using a commercially available Superoxide Dismutase Assay Kit (Cayman Chemical, Ann Arbor, MI, USA; Item No. 706002), according to the manufacturer’s instructions. The assay is based on inhibition of the reduction of a tetrazolium salt by superoxide radicals generated by xanthine oxidase. Absorbance was measured at 450 nm using a Multiskan™ FC microplate reader (Thermo Fisher Scientific Oy, Vantaa, Finland). Total protein concentration in the intestinal homogenates was determined using the Bradford method. SOD activity was normalized to the total protein content of each sample and expressed as U/mg protein.

### 4.6. Statistical Analysis

Quantitative data obtained from the immunofluorescence, RT-qPCR, and SOD activity analyses are presented as mean ± standard error of the mean (SEM). Prior to statistical analysis, normality of residuals was assessed using the Shapiro–Wilk test, and homogeneity of variances among groups was evaluated using Levene’s test. No significant deviations from normality or homogeneity of variance were detected (*p* > 0.05). Differences among the control (C), G1, and G2 groups were analysed using one-way analysis of variance (one-way ANOVA). When a significant overall group effect was detected, Tukey’s honestly significant difference (HSD) post hoc test was applied for pairwise comparisons between groups. For the immunofluorescence analysis, the percentage of SP-immunoreactive neurons calculated for each individual animal was treated as a single biological replicate (*n* = 5 per group). For the RT-qPCR analysis, technical replicates were averaged before statistical analysis, and individual animals were treated as biological replicates (*n* = 5 per group). For SOD activity measurements, each animal represented one biological replicate for each intestinal segment (*n* = 5 per group). For the analysis of the number of neurons per ganglion, 50 ganglia of each type (MP, OSP, and ISP) were evaluated in each experimental group, with 10 ganglia analysed per animal. The mean number of neurons per ganglion was calculated for each animal, and these animal-level means were used for statistical analysis (*n* = 5 per group). Statistical analyses were performed using Statistica software, version 13.3 (TIBCO Software Inc., Palo Alto, CA, USA). Differences were considered statistically significant at *p* < 0.05.

## 5. Conclusions

The present study demonstrates that oral exposure of pigs to low doses of glyphosate is associated with marked alterations in selected components of the enteric tachykinin system in the small intestine. Glyphosate exposure increased the percentage of substance P-immunoreactive enteric neurons in the duodenum, jejunum, and ileum and induced region-specific changes in tachykinin receptor gene expression. *TACR2* mRNA expression was increased in the duodenum in both glyphosate-exposed groups but decreased in the ileum at the higher glyphosate dose, whereas *TACR3* mRNA expression was decreased in the duodenum in both exposed groups and in the jejunum at the higher dose. *TACR1* mRNA expression remained unchanged. These findings indicate that the response of the enteric tachykinin system to glyphosate is complex and cannot be explained solely by changes in substance P immunoreactivity. The observed neurochemical remodelling may represent an adaptive response of the ENS to low-dose xenobiotic exposure; however, its functional consequences remain to be determined. Further studies integrating neurochemical, inflammatory, microbiota-related, and functional endpoints are required to clarify the mechanisms and physiological significance of these changes.

## Figures and Tables

**Figure 1:**
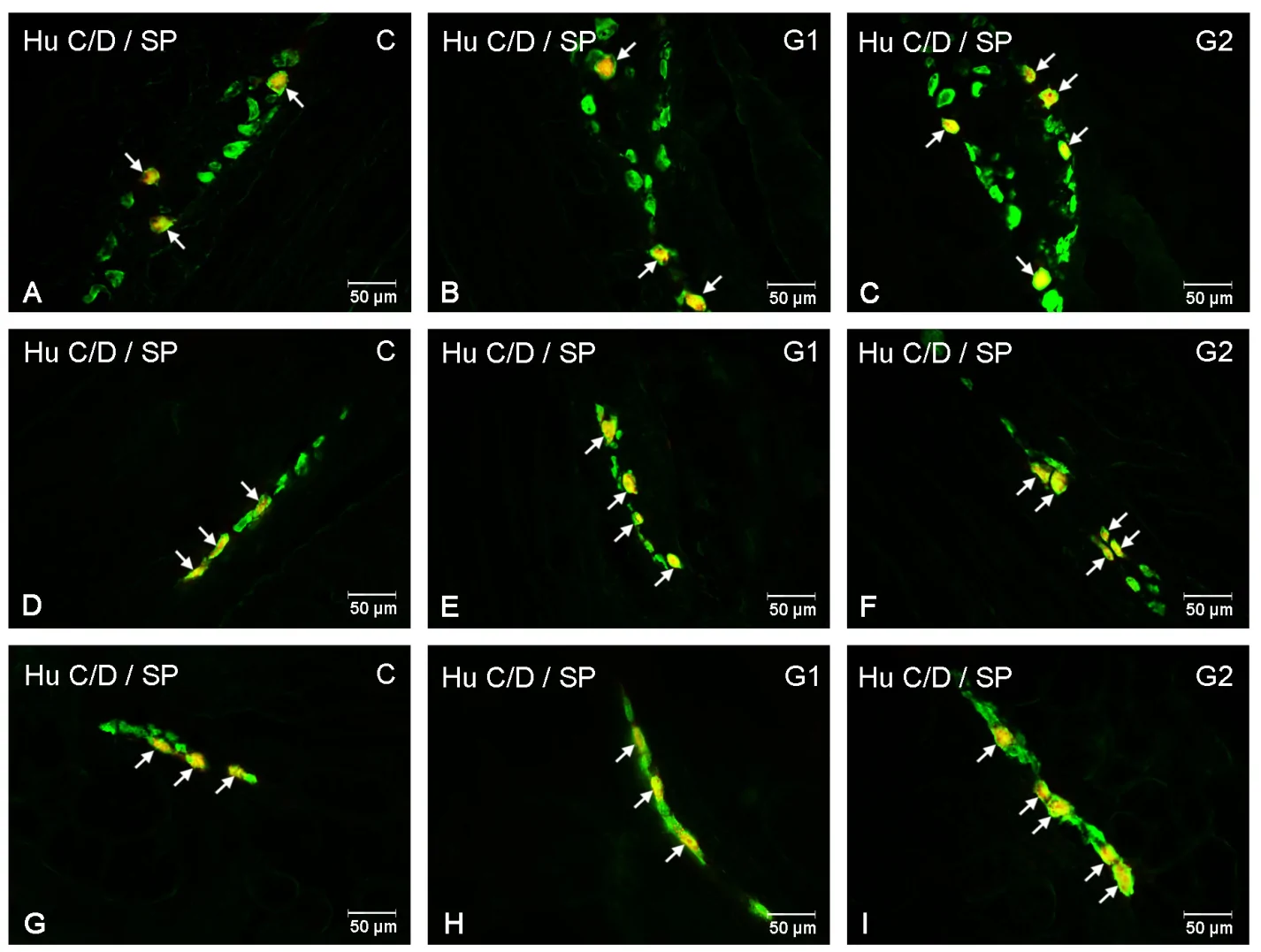
Representative double-immunofluorescence images illustrating substance P (SP)-immunoreactive enteric neurons in the porcine duodenum. Hu C/D, used as a pan-neuronal marker, is shown in green, whereas SP immunoreactivity is shown in red. The presented images are merged composites of the Hu C/D and SP fluorescence channels. SP-positive neurons are indicated by arrows. Panels (**A**–**C**) show the myenteric plexus (MP), panels (**D**–**F**) the outer submucosal plexus (OSP), and panels (**G**–**I**) the inner submucosal plexus (ISP). Control animals are shown in panels (**A**,**D**,**G**); animals receiving the lower glyphosate dose in panels (**B**,**E**,**H**); and animals receiving the higher glyphosate dose in panels (**C**,**F**,**I**).

**Figure 2:**
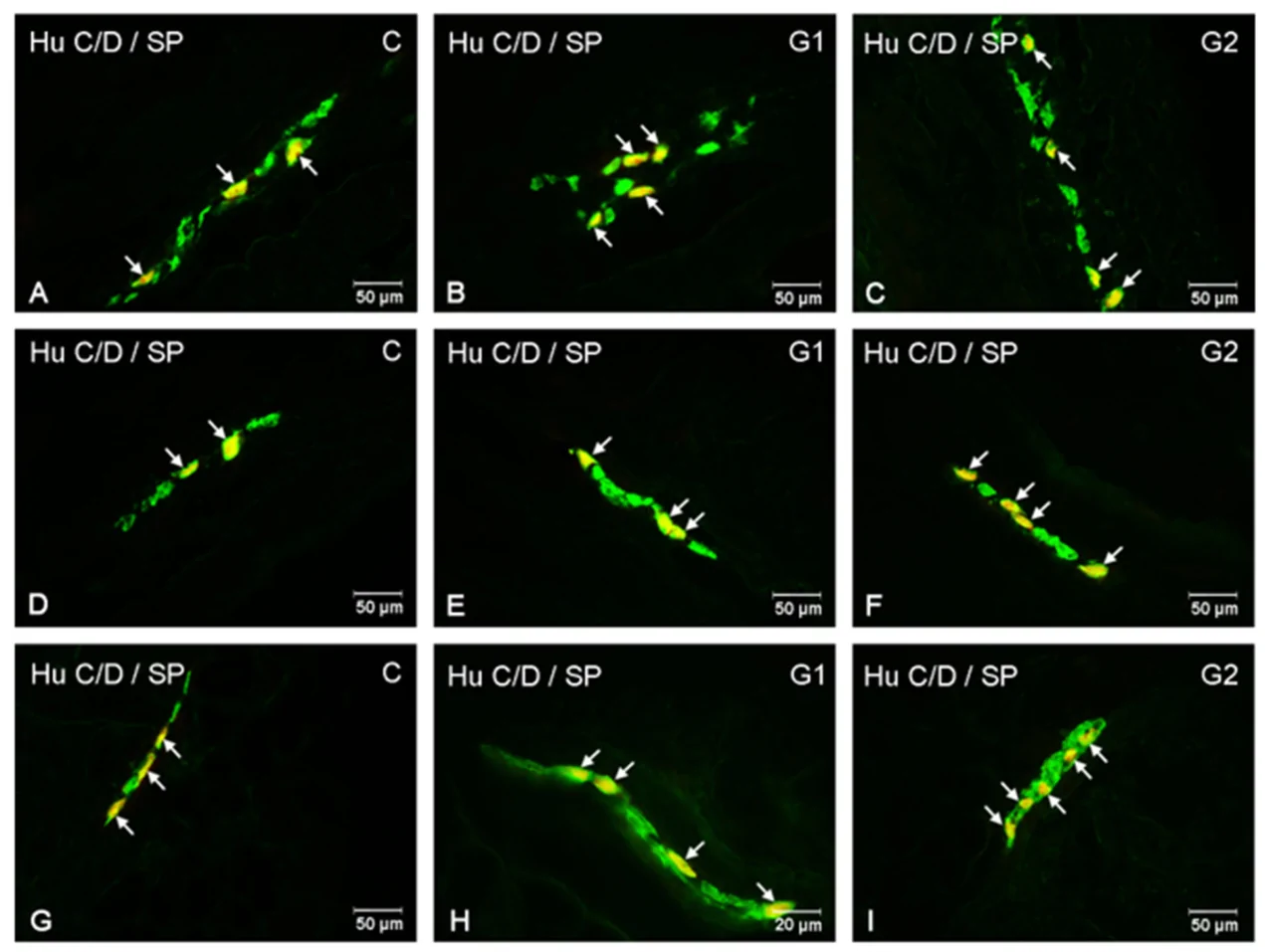
Representative double-immunofluorescence images illustrating substance P (SP)-immunoreactive enteric neurons in the porcine jejunum. Hu C/D, used as a pan-neuronal marker, is shown in green, whereas SP immunoreactivity is shown in red. The presented images are merged composites of the Hu C/D and SP fluorescence channels. SP-positive neurons are indicated by arrows. Panels (**A**–**C**) show the myenteric plexus (MP), panels (**D**–**F**) the outer submucosal plexus (OSP), and panels (**G**–**I**) the inner submucosal plexus (ISP). Control animals are shown in panels (**A**,**D**,**G**); animals receiving the lower glyphosate dose in panels (**B**,**E**,**H**); and animals receiving the higher glyphosate dose in panels (**C**,**F**,**I**).

**Figure 3:**
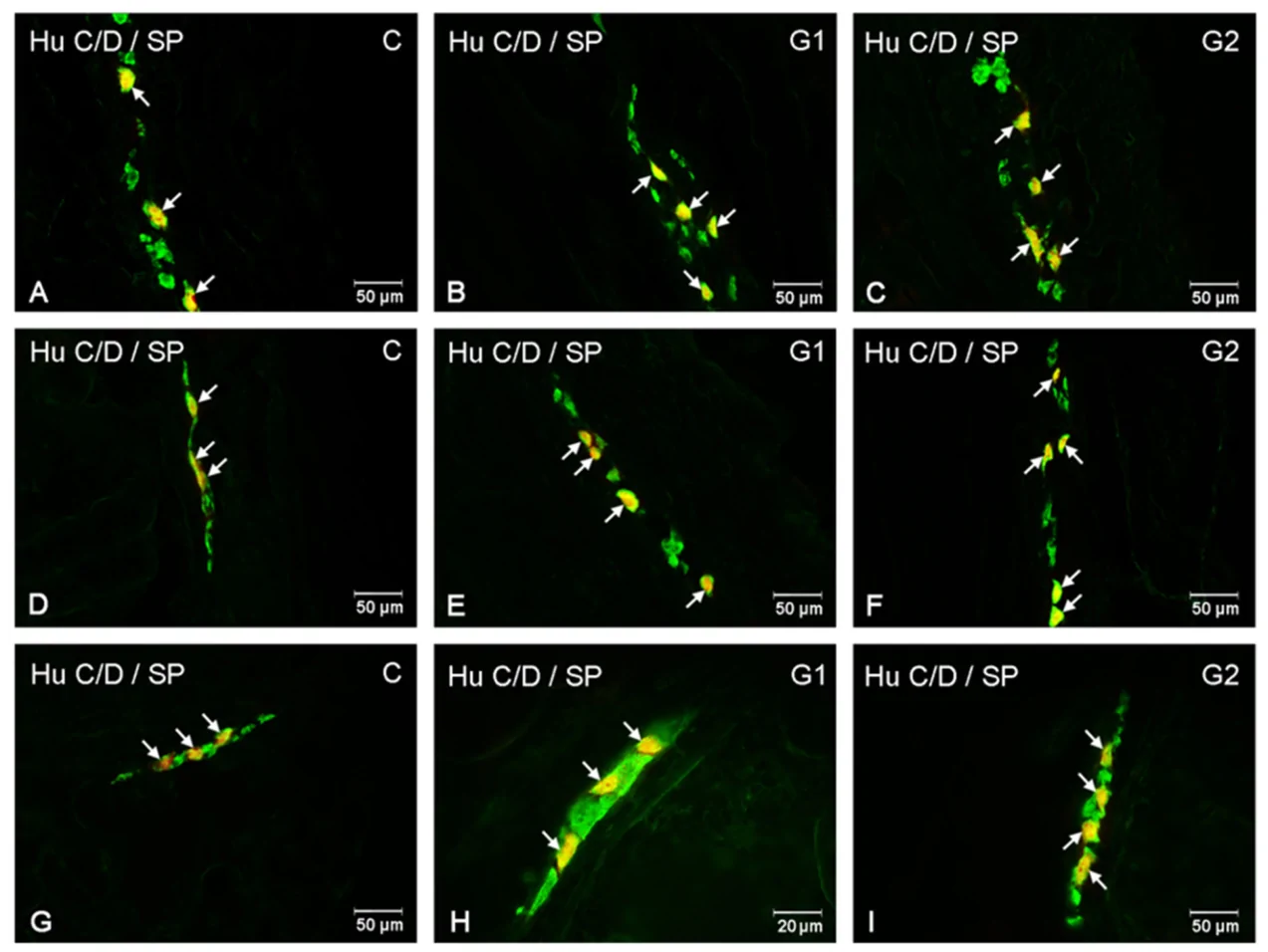
Representative double-immunofluorescence images illustrating substance P (SP)-immunoreactive enteric neurons in the porcine ileum. Hu C/D, used as a pan-neuronal marker, is shown in green, whereas SP immunoreactivity is shown in red. The presented images are merged composites of the Hu C/D and SP fluorescence channels. SP-positive neurons are indicated by arrows. Panels (**A**–**C**) show the myenteric plexus (MP), panels (**D**–**F**) the outer submucosal plexus (OSP), and panels (**G**–**I**) the inner submucosal plexus (ISP). Control animals are shown in panels (**A**,**D**,**G**); animals receiving the lower glyphosate dose in panels (**B**,**E**,**H**); and animals receiving the higher glyphosate dose in panels (**C**,**F**,**I**).

**Figure 4:**
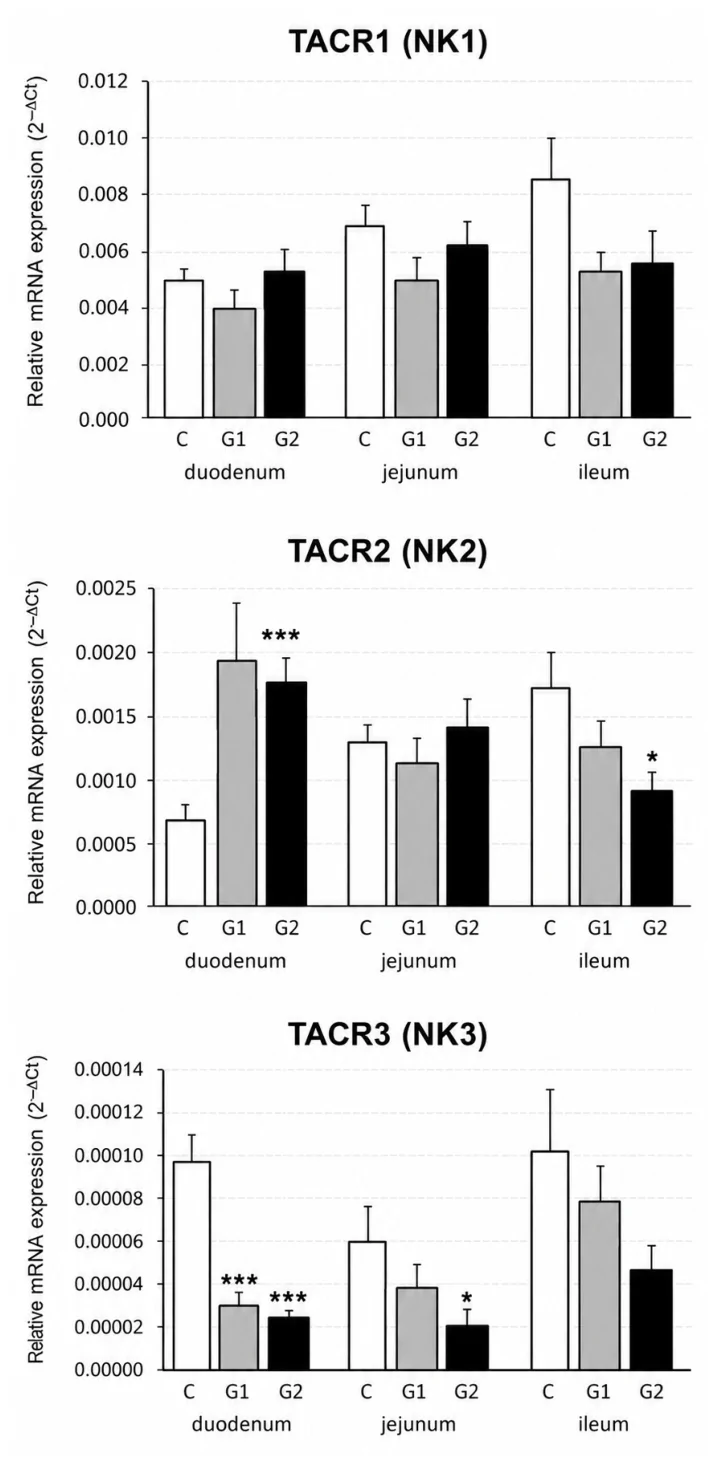
Relative expression of *TACR1* (NK1), *TACR2* (NK2), and *TACR3* (NK3) genes in the duodenum, jejunum, and ileum of pigs. Gene expression was quantified by RT-qPCR, with *GAPDH* used as the reference gene. Relative expression levels were calculated using the 2^−ΔCt^ method. White bars indicate the C group, grey bars indicate the G1 group, and black bars indicate the G2 group. Data are expressed as mean ± SEM. Statistical significance versus the C group is indicated as * *p* < 0.05 and *** *p* < 0.001.

**Figure 5:**
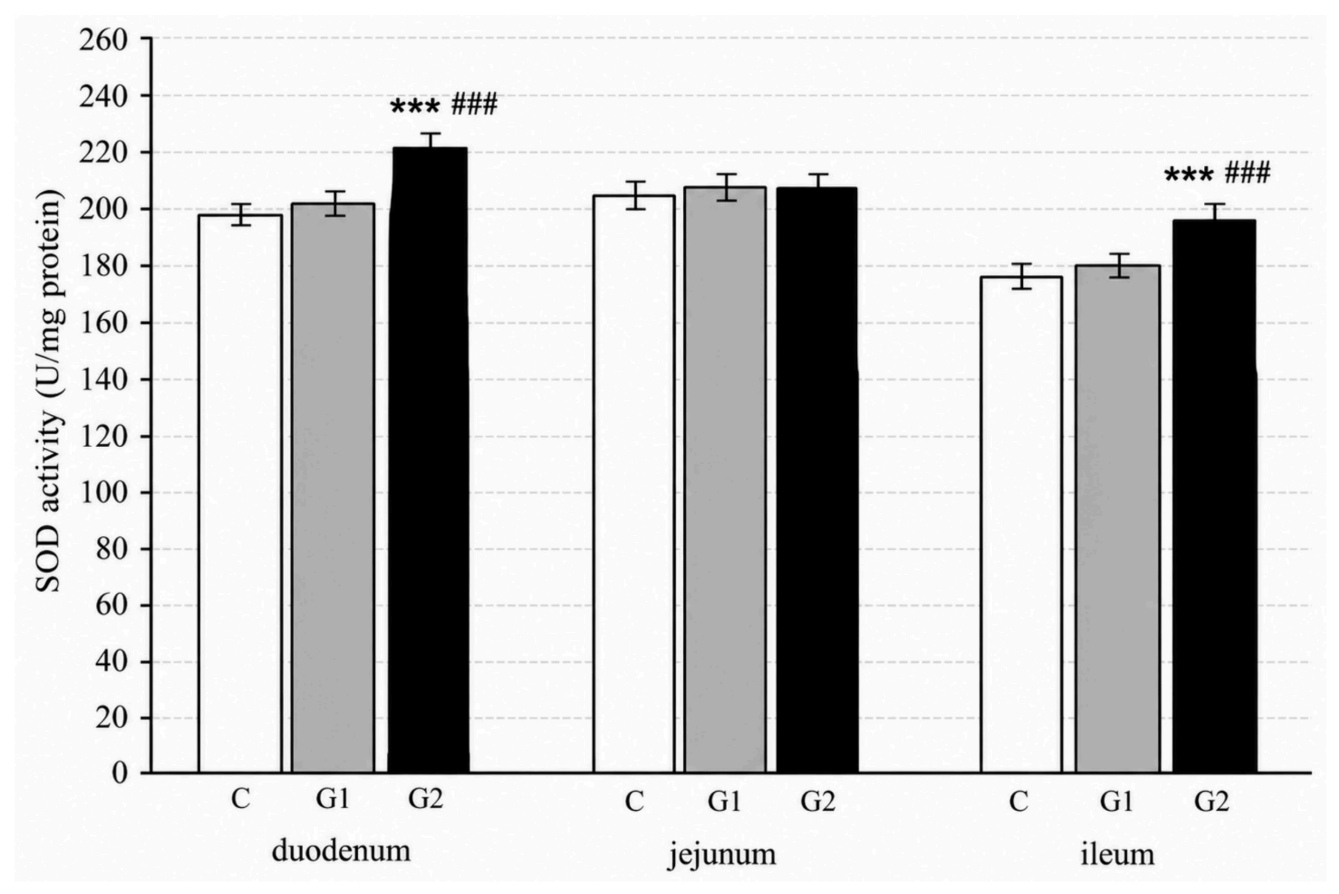
Superoxide dismutase (SOD) activity in the duodenum, jejunum, and ileum of pigs from the control group (C) and glyphosate-exposed groups (G1 and G2). White bars indicate the C group, grey bars indicate the G1 group, and black bars indicate the G2 group. Data are presented as mean ± SEM. *** *p* < 0.001 for G2 vs. C; ### *p* < 0.001 for G2 vs. G1.

**Table 1:** Percentage of SP-immunoreactive enteric neurons in the porcine small intestine.

	Myenteric Plexus			Outer Submucosal Plexus			Inner Submucosal Plexus		
	C Group	G1 Group	G2 Group	C Group	G1 Group	G2 Group	C Group	G1 Group	G2 Group
**Duodenum**	1.22 ± 0.15	1.55 ± 0.24	5.55 ± 0.47 (***, ###)	22.36 ± 1.12	26.65 ± 0.96	38.53 ± 1.44 (***, ###)	12.30 ± 0.89	20.21 ± 2.34 (*)	35.71 ± 1.90 (***, ###)
**Jejunum**	1.25 ± 0.20	3.27 ± 0.30 (***)	5.41 ± 0.26 (***, ###)	23.03 ± 1.43	31.25 ± 1.34 (**)	42.26 ± 1.04 (***, ###)	12.35 ± 0.70	16.36 ± 0.68 (*)	43.06 ± 1.21 (***, ###)
**Ileum**	1.01 ± 0.19	2.18 ± 0.31	6.11 ± 0.53 (***, ###)	15.63 ± 0.79	22.10 ± 1.52 (*)	35.93 ± 2.27 (***, ###)	20.22 ± 0.96	31.16 ± 1.84 (***)	43.11 ± 1.34 (***, ###)

C group, control group; G1 group, animals receiving glyphosate at a dose corresponding to the estimated maximum daily intake in Europe (0.05 mg/kg BW/day); G2 group, animals receiving glyphosate at a dose corresponding to the acceptable daily intake (ADI; 0.5 mg/kg BW/day). Data are presented as mean ± SEM. * *p* < 0.05, ** *p* < 0.01, and *** *p* < 0.001 vs. the control group; ### *p* < 0.001 vs. the G1 group.

**Table 2:** Effect of glyphosate exposure on the number of HuC/D-positive neurons per ganglion in the porcine small intestine.

		C	G1	G2	*p*-Value
**Duodenum**	**MP**	27.68 ± 2.32	26.74 ± 0.83	29.00 ± 1.79	0.567
	**OSP**	16.02 ± 1.10	15.04 ± 0.31	14.56 ± 0.45	0.364
	**ISP**	7.60 ± 0.32	8.20 ± 0.63	8.10 ± 0.55	0.689
**Jejunum**	**MP**	20.14 ± 0.92	17.16 ± 1.03	18.90 ± 1.83	0.314
	**OSP**	11.88 ± 0.67	11.26 ± 1.04	9.88 ± 0.91	0.302
	**ISP**	7.22 ± 0.30	7.60 ± 0.72	7.42 ± 0.20	0.848
**Ileum**	**MP**	26.22 ± 0.92	24.58 ± 2.00	23.62 ± 2.09	0.584
	**OSP**	12.68 ± 0.78	11.62 ± 1.16	12.64 ± 0.24	0.725
	**ISP**	8.20 ± 0.49	7.62 ± 0.54	7.92 ± 0.94	0.840

Data are presented as mean ± SEM (*n* = 5 animals per group). For each animal, the mean number of HuC/D-positive neurons per ganglion was calculated from 10 analysed ganglia. MP, myenteric plexus; OSP, outer submucosal plexus; ISP, inner submucosal plexus. No statistically significant differences were observed among the C, G1, and G2 groups (*p* > 0.05).

**Table 3:** Primer sequences used for RT-qPCR analysis.

Gene	Sequences of Primers	GenBank Accession Number
***GAPDH***	Forward: TTCCACCCACGGCAAGTT	NM_001206359.1
***GAPDH***	Reverse: GGCCTTTCCATTGATGACAAG	NM_001206359.1
***TACR1*** **(NK1)**	Forward: GCTGCCTCAATGACAGGTTTC	XM_021087317.1
***TACR1*** **(NK1)**	Reverse: GCCCCTCATAATCACCAGCA	XM_021087317.1
***TACR2*** **(NK2)**	Forward: GCCTCACGCTCTGGAGACGC	XM_001929073.7
***TACR2*** **(NK2)**	Reverse: GGCAGCCAGCAGATGGCGAA	XM_001929073.7
***TACR3*** **(NK3)**	Forward: GGCTGGCAATGAGTTCAACC	XM_003482466.4
***TACR3*** **(NK3)**	Reverse: AGCCAGCTCGAAATCTCTTGT	XM_003482466.4

## Data Availability

The original contributions presented in this study are included in the article. Further inquiries can be directed to the corresponding author.
